# Modulation of human platelet activation and in vivo vascular thrombosis by columbianadin: regulation by integrin α_IIb_β_3_ inside-out but not outside-in signals

**DOI:** 10.1186/s12929-020-0619-5

**Published:** 2020-05-06

**Authors:** Shaw-Min Hou, Chih-Wei Hsia, Cheng-Lin Tsai, Chih-Hsuan Hsia, Thanasekaran Jayakumar, Marappan Velusamy, Joen-Rong Sheu

**Affiliations:** 1 Department of Cardiovascular Center, Cathay General Hospital, No. 280 Renai Rd. Sec.4, Taipei, 106 Taiwan; 2 Division of Cardiovascular Surgery, Department of Surgery, School of Medicine, College of Medicine, Fu Jen Catholic University, No.510, Zhongzheng Rd, New Taipei City, 242 Taiwan; 3 Graduate Institute of Medical Sciences, College of Medicine, Taipei Medical University, 250 Wu-Hsing Street, Taipei, 110 Taiwan; 4 Graduate Institute of Metabolism and Obesity Sciences, Collage of Nutrition, Taipei Medical University, No. 250, Wu Hsing St, Taipei, 110 Taiwan; 5 Translational Medicine Center, Shin Kong Wu Ho-Su Memorial Hospital, No. 95, Wenchang Rd, Taipei, 111 Taiwan; 6 Department of Chemistry, North Eastern Hill University, Shillong, 793022 India

**Keywords:** CBN, Coumarin derivative, Platelet aggregation, Arterial thrombosis, Integrin α_IIb_β_3_

## Abstract

**Background:**

Columbianadin (CBN) is one of the main coumarin constituents isolated from *Angelica pubescens*. The pharmacological value of CBN is well demonstrated, especially in the prevention of several cancers and analgesic activity. A striking therapeutic target for arterial thrombosis is inhibition of platelet activation because platelet activation significantly contributes to these diseases. The current study examined the influence of CBN on human platelet activation in vitro and vascular thrombotic formation in vivo.

**Methods:**

Aggregometry, immunoblotting, immunoprecipitation, confocal microscopic analysis, fibrin clot retraction, and thrombogenic animals were used in this study.

**Results:**

CBN markedly inhibited platelet aggregation in washed human platelets stimulated only by collagen, but was not effective in platelets stimulated by other agonists such as thrombin, arachidonic acid, and U46619. CBN evidently inhibited ATP release, intracellular ([Ca^2+^]i) mobilization, and P-selectin expression. It also inhibited the phosphorylation of phospholipase C (PLC)γ2, protein kinase C (PKC), Akt (protein kinase B), and mitogen-activated protein kinases (MAPKs; extracellular signal-regulated kinase [ERK] 1/2 and c-Jun N-terminal kinase [JNK] 1/2, but not p38 MAPK) in collagen-activated platelets. Neither SQ22536, an adenylate cyclase inhibitor, nor ODQ, a guanylate cyclase inhibitor, reversed the CBN-mediated inhibition of platelet aggregation. CBN had no significant effect in triggering vasodilator-stimulated phosphoprotein phosphorylation. Moreover, it markedly hindered integrin α_IIb_β_3_ activation by interfering with the binding of PAC-1; nevertheless, it had no influences on integrin α_IIb_β_3_-mediated outside-in signaling such as adhesion number and spreading area of platelets on immobilized fibrinogen as well as thrombin-stimulated fibrin clot retraction. Additionally, CBN did not attenuate FITC-triflavin binding or phosphorylation of proteins, such as integrin β_3_, Src, and focal adhesion kinase, in platelets spreading on immobilized fibrinogen. In experimental mice, CBN increased the occlusion time of thrombotic platelet plug formation.

**Conclusion:**

This study demonstrated that CBN exhibits an exceptional activity against platelet activation through inhibition of the PLCγ2-PKC cascade, subsequently suppressing the activation of Akt and ERKs/JNKs and influencing platelet aggregation. Consequently, this work provides solid evidence and considers that CBN has the potential to serve as a therapeutic agent for the treatment of thromboembolic disorders.

## Introduction

Arterial thrombosis can lead to the development of cardiovascular diseases (CVDs) such as myocardial infarction, atherosclerosis, and even ischemic stroke. When vascular subendothelial connective tissues are exposed due to injury, platelets move, adhere at the site of injury, and subsequently initiate the vascular thrombosis. Collagen contained in the basement membrane induces a change in shape from discoid to spheroid with pseudopodic projections of platelets. The combination of platelet secretion from the granules contain ADP/ATP, Ca^2+^, and fibrinogen, allows engagement of platelet receptors initiates intra-platelet signaling pathways, which activates platelet integrin α_IIb_β_3_ and enables platelet aggregation [1]. In resting platelets, integrin α_IIb_β_3_ exists in a low activation state and is unable to interact with its specific ligands such as fibrinogen, fibronectin, and von Willebrand factor. Platelet activation stimulated by various agonists induces a conformational change in integrin α_IIb_β_3_, enabling it to bind to its ligands, resulting in the onset of platelet aggregation; this process is known as inside-out signal transduction [1]. Moreover, the binding of fibrinogen to activated integrin α_IIb_β_3_ initiates a series of intracellular signaling events, such as tyrosine phosphorylation of numerous proteins and cytoskeleton reorganization; this process is referred to as outside-in signaling [1]. These outside-in reactions, originating in the integrin α_IIb_β_3_ bound to fibrinogen, are required for maximal secretion, procoagulation, and clot retraction [1].

**Fig. 1:**
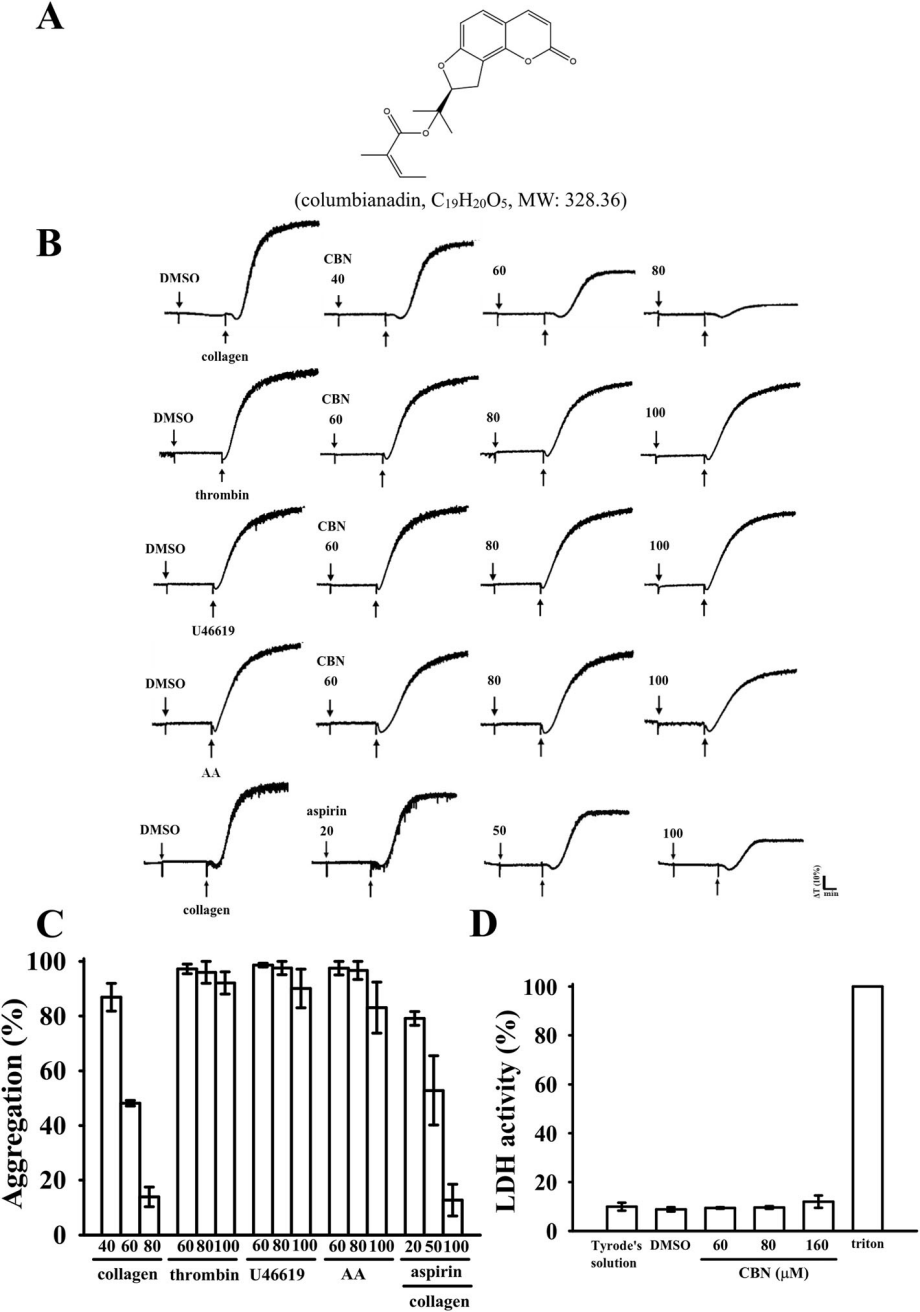
Inhibitory activities of columbianadin (CBN) on platelet aggregation and cytotoxicity stimulated by agonists. Washed human platelets (3.6 × 10^8^ cells/mL) were preincubated with a solvent control (0.1% DMSO), CBN (40–100 μM) (**a**, chemical structure) or aspirin (20–100 μM) and subsequently treated with 1 μg/mL of collagen, 0.01 U/mL of thrombin, 1 μM U46619, or 60 μM of arachidonic acid (AA) to stimulate platelet aggregation (**b**). Concentration–response histograms of CBN in inhibition of platelet aggregation (%) (**c**). To assess the cytotoxicity (**d**), platelets were preincubated with the solvent control (0.1% DMSO) or CBN (60, 80, and 160 μM) for 20 min, and a 10-μL aliquot of the supernatant was deposited on a Fuji Dri-Chem slide LDH-PIII. Data (**c** and **d**) are presented as the means ± SEM (*n* = 4)

Columbianadin (CBN; Fig. 1a) is a natural coumarin-type compound isolated from the root of *Angelica pubescens* Maxim. *f. biserrata* Shan *et* Yuan, which is mainly used to treat rheumatism, spasm, and headache in clinics, according to Chinese Pharmacopoeia [2]. Many coumarin derivatives are isolated from *A. pubescens*, of which CBN is one of the main bioactive constituents. CBN has attracted considerable attention due to its pharmacological properties, such as prevention of several types of cancers (i.e., human leukemia, bladder carcinoma, and colon cancer). It can effectively suppress the growth of colon cancer cells by inducing apoptosis at low concentrations (~ 25 μM) and necroptosis at high concentrations (50 μM). The induction of apoptosis by CBN is correlated with the modulation of caspase-9, caspase-3, Bax, Bcl-2, Bim, and Bid, and the induction of necroptosis is related to receptor-interacting protein kinase-3 and caspase-8 [2]. CBN also possesses analgesic properties. Moreover, it causes the inhibition of inflammatory responses, which markedly inhibited edema and the vascular permeability in mice and reduced the inflammatory response in LPS-induced lung injury through the downregulation of inducible nitric oxide synthase in mice [3, 4].

CBN was also preliminary reported to exhibit antiplatelet activity stimulated by ADP in rat platelets [5]; however, the effects and mechanisms of this compound on human platelets have not been investigated. Our initial screening exhibited that CBN significantly inhibits aggregation in human platelets. This result inspired us to conduct a thorough investigation on the influence of CBN on human platelets to support the scientific rationale for its clinical use (i.e., to treat CVDs).

## Materials and methods

### Materials

Collagen (type I), luciferin-luciferase, arachidonic acid (AA), U46619, ADP, fibrinogen, phorbol-12,13-dibutyrate (PDBu), heparin, prostaglandin E_1_ (PGE_1_), fluorescein isothiocyanate (FITC)-phalloidin, nitroglycerin (NTG), aspirin and thrombin were purchased from Sigma (St. Louis, MO, USA). CBN (> 98%) was obtained from the ChemFaces Biohem (Wuhan, Hubei, China). Fura 2-AM was purchased from Molecular Probes (Eugene, OR, USA). The anti-phospho-p38 mitogen-activated protein kinase (MAPK) Ser182, anti-integrin β_3_ monoclonal antibodies (mAbs), and anti-phospho-integrin β_3_ (Tyr759) polyclonal antibody (pAb) were purchased from Santa Cruz (Santa Cruz, CA, USA). The anti-p38 MAPK, anti-phospho-c-Jun N-terminal kinase (JNK) (Thr183/Tyr185), anti-phospho-Src family (Tyr416), and anti-phospho-focal adhesion kinase (FAK) (Tyr397) mAbs and anti-phospholipase C (PLC)γ2, anti-phospho (Tyr759) PLCγ2, anti-phospho-p44/p42 extracellular signal-regulated kinase (ERK) (Thr202/Tyr204), and anti-Src family pAbs were purchased from Cell Signaling (Beverly, MA, USA). The anti-phospho-protein kinase B (Akt; Ser473) and anti-Akt mAbs were purchased from Biovision (Mountain View, CA, USA). An anti-FAK pAb was obtained from Millipore (Billerica, MA, USA). The anti-α-tubulin mAb was purchased from NeoMarkers (Fremont, CA, USA). FITC-anti-human CD42P (P-selectin) and FITC-anti-human CD41/CD61 (PAC-1) mAbs were obtained from BioLegend (San Diego, CA, USA). Protein G Mag Sepharose Xtra Beads were purchased from GE Healthcare (Uppsala, Sweden). A Hybond-P Polyvinylidene difluoride membrane, an enhanced chemiluminescence Western blotting detection reagent, horseradish peroxidase-conjugated donkey anti-rabbit immunoglobulin G (IgG), and sheep anti-mouse IgG were purchased from Amersham (Buckinghamshire, UK). CBN suspension was prepared in 0.1% dimethyl sulfoxide (DMSO) and stored at 4 °C.

### Platelet preparation, aggregation, and ATP release

This study complied with the directives of the Helsinki Declaration and was approved by the Institutional Review Board of Taipei Medical University. Informed consent was obtained from all human volunteers who participated in this study. Washed human platelets (3.6 × 10^8^ cells/mL) were prepared as described previously [6], and CBN (10–100 μM), aspirin (20–100 μM) or solvent control (0.1% DMSO) was incubated with the platelets for 3 min before stimulation. ATP release was measured using Hitachi Spectrometer F-7000 (Tokyo, Japan) according to the manufacturer’s protocol.

### Intracellular [Ca^2+^]i mobilization and lactate dehydrogenase assays

To measure the intracellular calcium [Ca^2+^]i, citrated whole blood was centrifuged, and the supernatant was incubated with 5 μM Fura 2-AM, which was then measured using a Hitachi Spectrometer F-7000 (Tokyo, Japan). [Ca^2+^]i was measured at excitation wavelengths of 340 and 380 nm and an emission wavelength of 500 nm [7]. Furthermore, cytotoxic effect was examined by determining the level of lactate dehydrogenase (LDH). Washed platelets were preincubated with CBN (60, 80, and 160 μM) or 0.1% DMSO for 20 min at 37 °C. An aliquot of the supernatant (10 μL) was deposited on a Fuji Dri-Chem slide LDH-PIII (Fuji, Tokyo, Japan), and the absorbance was read using a spectrophotometer (UV-160; Shimadzu, Japan). The maximal value of LDH was observed in triton-treated platelets.

### Surface P-selectin expression and integrin α_IIb_β_3_ activation

Briefly, washed platelets were preincubated with CBN (60 and 80 μM) and the FITC-conjugated anti-P-selectin mAb (2 μg/mL) or PAC-1 mAb (2 μg/mL) for 3 min and then stimulated by collagen (1 μg/mL). For other experiments, fluorescence-conjugated triflavin, a specific integrin α_IIb_β_3_ antagonist, was prepared as described previously [8]. The final concentration of FITC-triflavin was adjusted to 1 mg/mL. Washed platelets were preincubated with EDTA (2 mM), CBN (60 and 80 μM) or solvent control (0.1% DMSO), followed by the addition of FITC-triflavin (2 μg/mL) for 3 min. The suspensions were then assayed for fluorescein-labeled platelets on a flow cytometer (FAC Scan system, Becton Dickinson, San Jose, CA, USA). Data were collected from 50,000 platelets per experimental group, and the platelets were identified based on their characteristic forward and orthogonal light-scattering profiles. All experiments were repeated at least four times to ensure reproducibility.

### Confocal microscopic analysis of platelet adhesion and spreading on immobilized fibrinogen

Platelet spreading on immobilized fibrinogen was analyzed as described previously [9]. In brief, platelets were stained with FITC-labeled phalloidin and visualized with a Leica TCS SP5 microscope equipped with a 100×, 1.40 NA oil immersion objective (Leica, Wetzlar, Germany). The number of platelet adhesion events and the platelet spreading surface area were determined using the NIH ImageJ software (NIH, Bethesda, MD; http://rsbweb.nih.gov/ij/).

### Platelet-mediated fibrin clot retraction

Washed platelets were suspended in Tyrode’s solution containing 2 mg/mL fibrinogen and 1 mM CaCl_2_ in tubes designed for aggregation [10]. The platelet suspensions were preincubated in CBN (60 and 80 μM) or 0.1% DMSO for 3 min prior to thrombin (0.01 U/mL)-induced clot retraction without stirring. The reaction was photographed at 15 and 30 min, respectively.

### Immunoblotting

Washed platelets (1.2 × 10^9^ cells/mL) were preincubated with CBN (60 and 80 μM) or 0.1% DMSO, and collagen was subsequently added to trigger activation. The platelet suspensions were lysed and separated on a 12% SDS-PAGE. Several proteins were detected by specific primary antibodies. Respective quantitative results were obtained by quantifying the optical density of protein bands using a video densitometer and Bio-profil Biolight software, Version V2000.01 (VilberLourmat, Marne-la-Vallée, France).

### Immunoprecipitation

Dishes (6-cm diameter) were precoated with fibrinogen (100 μg/mL) overnight and then blocked with 1% BSA. Washed platelets were preincubated with CBN (80 μM) or the solvent control (0.1% DMSO) for 3 min and then allowed to spread on dishes for 60 min. The platelets were lysed and centrifuged; subsequently, Protein G Mag Sepharose Xtra beads (10 μL) was added and the platelets were incubated with the anti-integrin β_3_ mAb (1 μg/mL) for immunoblotting as described previously.

### Vascular thrombus formation in mouse mesenteric microvessels irradiated by sodium fluorescein

The method applied to the thrombogenic animal model in this experiment conformed to the Guide for the Care and Use of Laboratory Animals (8th edition, 2011), and we received an affidavit of approval for the animal use protocol from Taipei Medical University. In brief, external jugular veins of mice (6 weeks old) were cannulated with a polyethylene (PE)-10 tube for administration of the sodium fluorescein (15 μg/kg) and CBN (5 and 10 mg/kg) intravenously as described previously [11]. Venules (30–40 μm) were irradiated with wavelengths of < 520 nm to produce a microthrombus, and the time required for the thrombus to occlude the microvessel (occlusion time) was recorded.

### ADP-induced acute pulmonary thromboembolism in mice

Acute pulmonary thromboembolism was induced according to a previously described method [12]. Various doses of CBN (5 and 10 mg/kg), aspirin (20 mg/kg) or solvent control (0.1% DMSO) (all in 50 μL) were administered through intraperitoneal injection to mice. After 5 min, ADP (0.7 mg/g) was injected into the tail vein. The mortality of mice in each group within 10 min after injection was determined.

### Statistical analysis

The results are expressed as the means ± SEM and are accompanied by the number of observations (*n*). *n* refers to the number of experiments, and each experiment was conducted using different blood donors. The unpaired Student’s *t* test or analysis of variance was used to determine the significant differences among the groups. When this analysis indicated significant differences, the groups were compared using the Student–Newman–Keuls method. Statistical significance was set at *p <* 0.05.

## Results

### Inhibitory activities of CBN in platelet aggregation stimulated by various agonists

CBN (M.W. 328.36; C_19_H_20_O_5_) is a coumarin derivative of (Z)-2-methyl-2-butenoic acid 2-[(8S)-2-oxo-8, 9-dihydrofuro[2,3-h] [1] benzopyran-8-yl]propan-2-yl ester (Fig. 1a). Li et al. [5] reported that CBN significantly inhibits rat platelet aggregation stimulated by ADP. However, no other study has reported this effect of CBN. In the current study, CBN (40–80 μM) more selectively inhibited human platelet aggregation stimulated by collagen (1 μg/mL) than AA, thrombin, or U46619 (a thromboxane A_2_ receptor agonist). Although CBN slightly but no significantly inhibited platelet aggregations even at concentrations up to 100 μM (Fig. 1b–c). The 50% inhibitory concentration (IC_50_) of CBN for collagen-induced platelet aggregation was approximated at 60 μM (Fig. 1c). Moreover, aspirin (20, 50, and 100 μM) concentration-dependently inhibited platelet aggregation stimulated by collagen (1 μg/mL), and its IC_50_ value was approximated at 70 μM (Fig. 1b and c). The solvent control (0.1% DMSO) did not exert any significant effects on platelet aggregation (Fig. 1b). The LDH study revealed that CBN (60, 80, and 160 μM) did not alter LDH release or have any cytotoxic effects on platelets (Fig. 1d). This result revealed that CBN did not affect platelet permeability or induce platelet cytolysis.

### Regulatory profiles of CBN in ATP release, [Ca^2+^] mobilization, and P-selectin expression

**Fig. 2:**
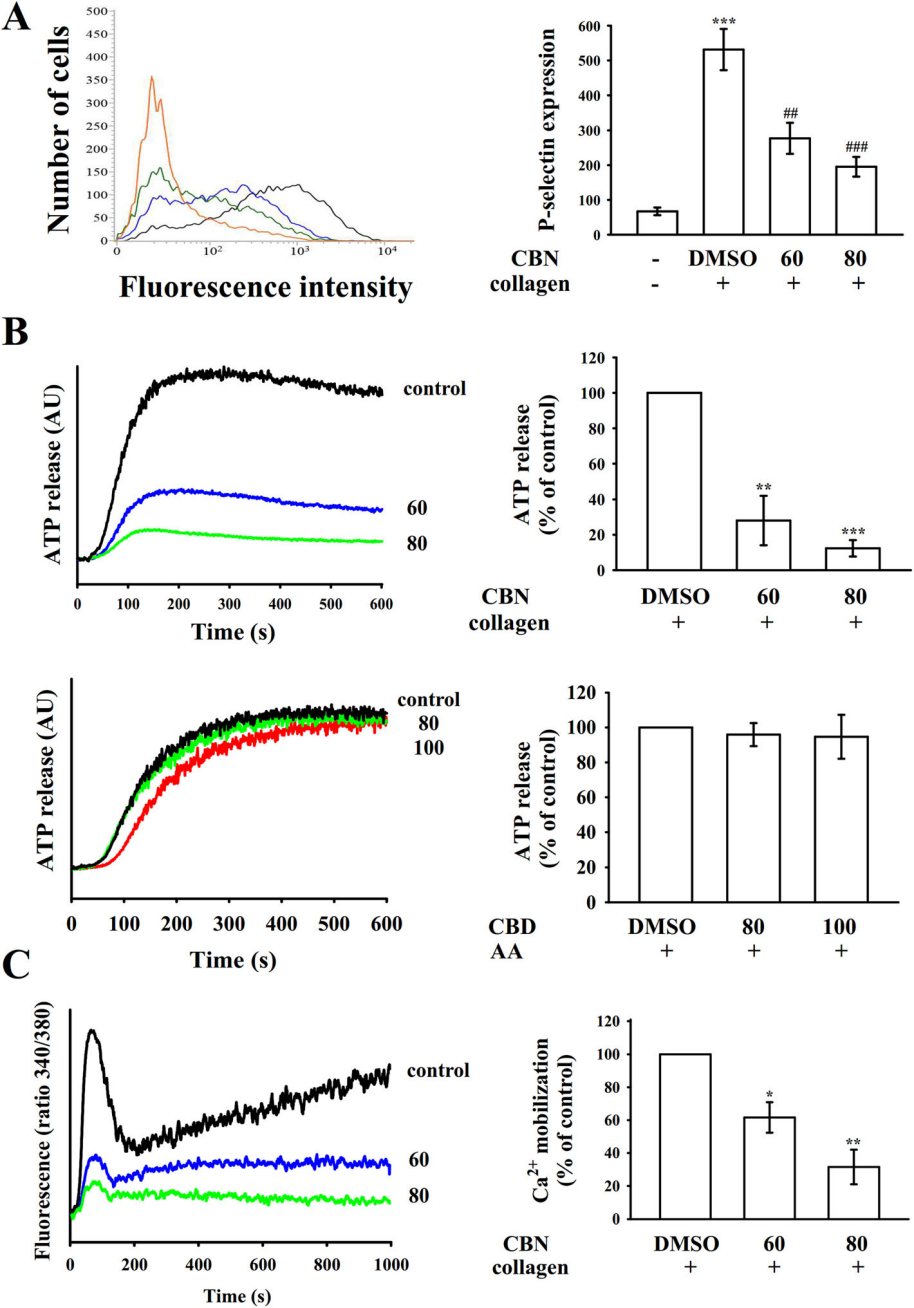
Effectiveness of columbianadin (CBN) on surface P-selectin expression, ATP releases, and relative [Ca^2+^]i mobilization in human platelets. Washed platelets (3.6 × 10^8^ cells/mL) were preincubated with the solvent control (0.1% DMSO) or CBN (60 and 80 μM), followed by the addition of collagen (1 μg/mL) or arachidonic acid (AA; 60 μM) to trigger (**a**) surface P-selectin expression (resting control, red line; collagen-activated, black line; CBN 60 μM, blue line; CBN 80 μM, green line), (**b**) ATP release (AU; arbitrary unit), and (**c**) relative [Ca^2+^]i mobilization, as described in the Materials and methods section. The corresponding statistical data are presented in the right panel of each figure. Data are presented as means ± SEM (*n* = 4). ^*^p < 0.05, ^**^p < 0.01, and ^***^p < 0.001 compared with the resting control; ^##^*p* < 0.01 and ^###^*p* < 0.001, compared with the 0.1% DMSO-treated group

Platelet activation is associated with the release of granular contents (e.g., P-selectin from α-granules, ADP/ATP and Ca^2+^ from dense granules), causing amplification of platelet activation. P-selectin is located on the inner wall of α-granules in the resting platelets, and the inner walls of the granules are exposed on the outside of the cells after activation [13]. CBN evidently reduced collagen-stimulated surface P-selectin expression (resting control, 67.0 ± 11.1; collagen-activated, 531.3 ± 59.3; CBN 60 μM, 277.0 ± 44.6; and CBN 80 μM, 195.3 ± 28.4; *n* = 4; Fig. 2a). As presented in Fig. 2b, CBN diminished the ATP-release reaction stimulated by collagen (1 μg/mL), but not by AA (60 μM). Moreover, collagen triggered a relative increase in [Ca^2+^]i, which was markedly reduced in the presence of CBN (60 and 80 μM) approximately by 40 and 70%, respectively (Fig. 2c).

### Effectiveness of CBN in PLCγ2/protein kinase C (PKC) and Akt activation

**Fig. 3:**
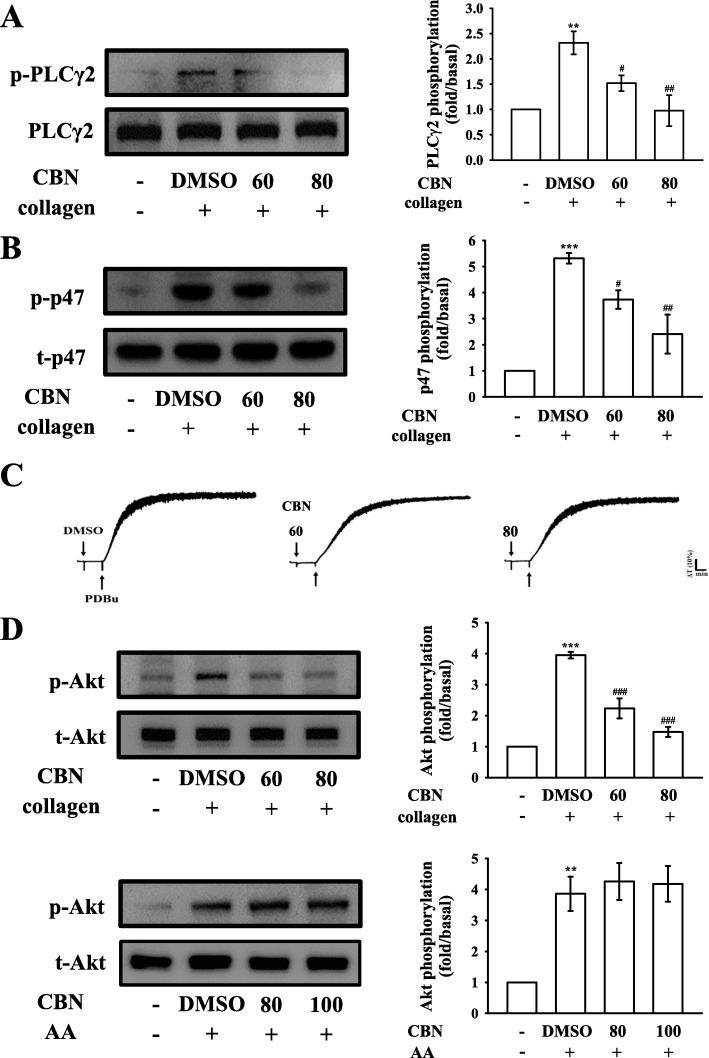
Regulatory effects of columbianadin (CBN) on PLCγ2/PKC and Akt phosphorylation in platelets. Washed platelets were preincubated with a solvent control (0.1% DMSO) or CBN (60 and 80 μM) and subsequently treated with collagen (1 μg/mL), PDBu (150 nM) or arachidonic acid (AA; 60 μM) to trigger either (**a**) PLCγ2, (**b**) PKC (p47) activation or (**c**) platelet aggregation, and (**d**) Akt phosphorylation. Profiles in (**c**) are representative of four independent experiments. Data are presented as the means ± SEM (*n =* 4). ^**^p < 0.01 and ^***^p < 0.001, compared with the resting platelets; ^#^*p <* 0.05, ^##^*p <* 0.01, and ^###^*p <* 0.001, compared with the 0.1% DMSO-treated group

PLC, belonging to a family of kinases, hydrolyzes phosphatidylinositol 4,5-bisphosphate [PI(4,5)P2] to produce two important secondary messengers, diacylglycerol (DAG) and inositol trisphosphate (IP_3_). DAG activates the PKC-inducing pleckstrin phosphorylation (47 kD protein) and ATP release in activated platelets; IP_3_ elevates calcium influx [14]. CBN (60 and 80 μM) obviously diminished the PLCγ2 phosphorylation and PKC activation in collagen-activated platelets (Figs. 3a–b). CBN did not significantly reduce PDBu (PKC activator)-induced platelet aggregation at 60 or 80 μM (Fig. 3c), indicating that CBN did not directly influence PKC activation. In addition, the Akt (Ser-Thr kinase) pathway or termed PI3K (phosphatidylinositol 3-kinase)-Akt pathway mediates downstream responses, including cell survival, growth, and platelet activation [15]. CBN (60–100 μM) markedly diminished the phosphorylation of Akt stimulated by collagen, but not by AA (Fig. 3d).

### Regulatory activities of CBN in MAPK activation

**Fig. 4:**
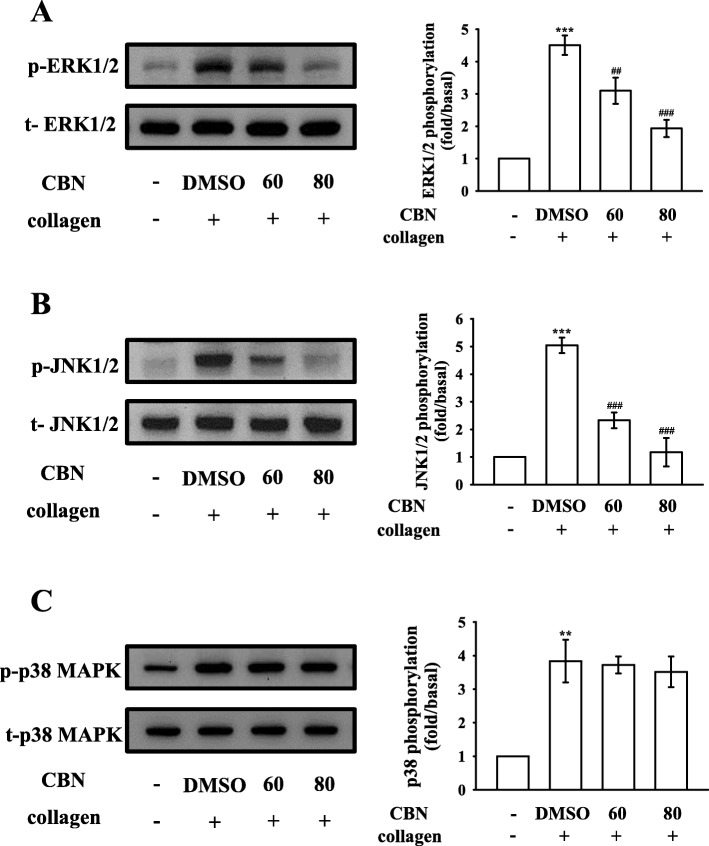
Effect of columbianadin (CBN) on ERK1/2, JNK1/2, and p38 MAPK phosphorylation in collagen-activated platelets. Washed platelets were preincubated with a solvent control (0.1% DMSO) or CBN (60 and 80 μM) and subsequently treated with collagen (1 μg/mL) for immunoblotting the (**a**) ERK1/2, (**b**) JNK1/2, and (**c**) p38 MAPK phosphorylation. Data are presented as the means ± SEM (n *=* 4). ^**^p < 0.01 and ^***^p < 0.001, compared with the resting platelets; ^##^*p <* 0.01 and ^###^*p <* 0.001, compared with the 0.1% DMSO-treated group

The inhibitory mechanisms of CBN in platelet activation were examined by investigating several MAPK signals, such as ERK1/2, JNK1/2, and p38 MAPK, which control major cellular functions, including proliferation, differentiation, and platelet activation [16]. Interestingly, CBN inhibited the phosphorylation of either ERK1/2 (Fig. 4a) or JNK1/2 (Fig. 4b), but not p38 MAPK (Fig. 4c), indicating that inhibition of the ERK1/2 and JNK1/2 signaling may be crucially involved in CBN-mediated inhibitory mechanisms.

### Roles of CBN in intracellular cyclic nucleotide formation and integrin α_IIb_β_3_ activation

**Fig. 5:**
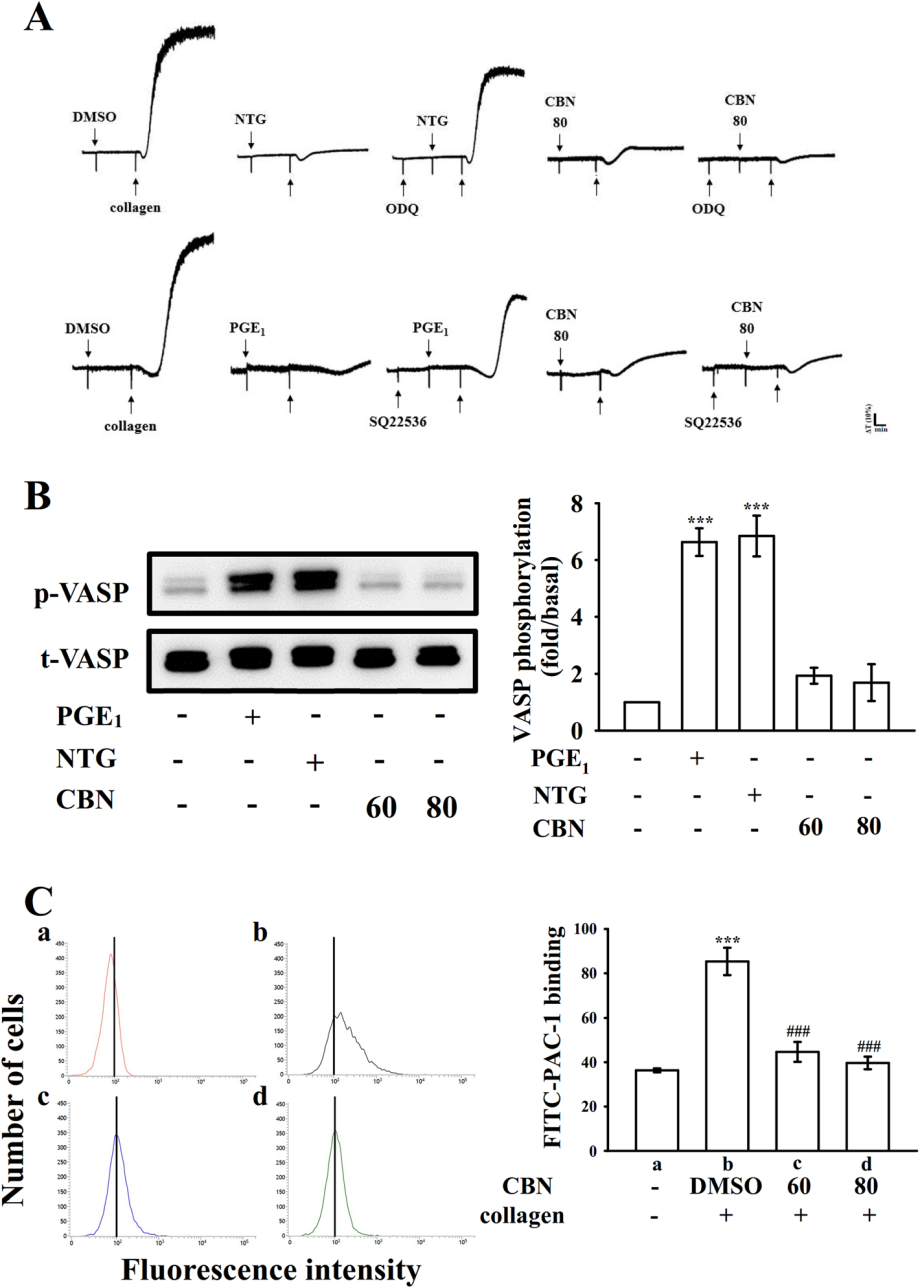
Effect of columbianadin (CBN) in cyclic nucleotides and vasodilator-stimulated phosphoprotein (VASP) phosphorylation as well as integrin α_IIb_β_3_ activation. Washed platelets (3.6 × 10^8^ cells/mL) were preincubated with (**a**) prostaglandin E_1_ (PGE_1_; 1 μM), nitroglycerin (NTG; 10 μM), or CBN (80 μM) in the presence of SQ22536 (100 μM) or ODQ (10 μM) for 3 min before addition of collagen (1 μg/mL) to trigger platelet aggregation. (**b**) For immunoblotting the VASP phosphorylation, washed platelets were stimulated with PGE_1_ (1 μM), NTG (10 μM), or CBN (60 and 80 μM). (**c**) For flow cytometry analysis, resting platelets (*a*, red line) or platelets were preincubated with the solvent control (*b*, 0.1% DMSO, black line) or CBN (*c*, 60 μM, blue line; *d*, 80 μM, green line) and FITC-conjugated anti-PAC-1 mAb (2 μg/mL) was added before the addition of collagen. Profiles in (**a**) are representative of four independent experiments. Data are presented as the means ± SEM (*n* = 4). ^***^p < 0.001, compared with the resting group; ^###^*p <* 0.001, compared with the 0.1% DMSO-treated group

**Fig. 6:**
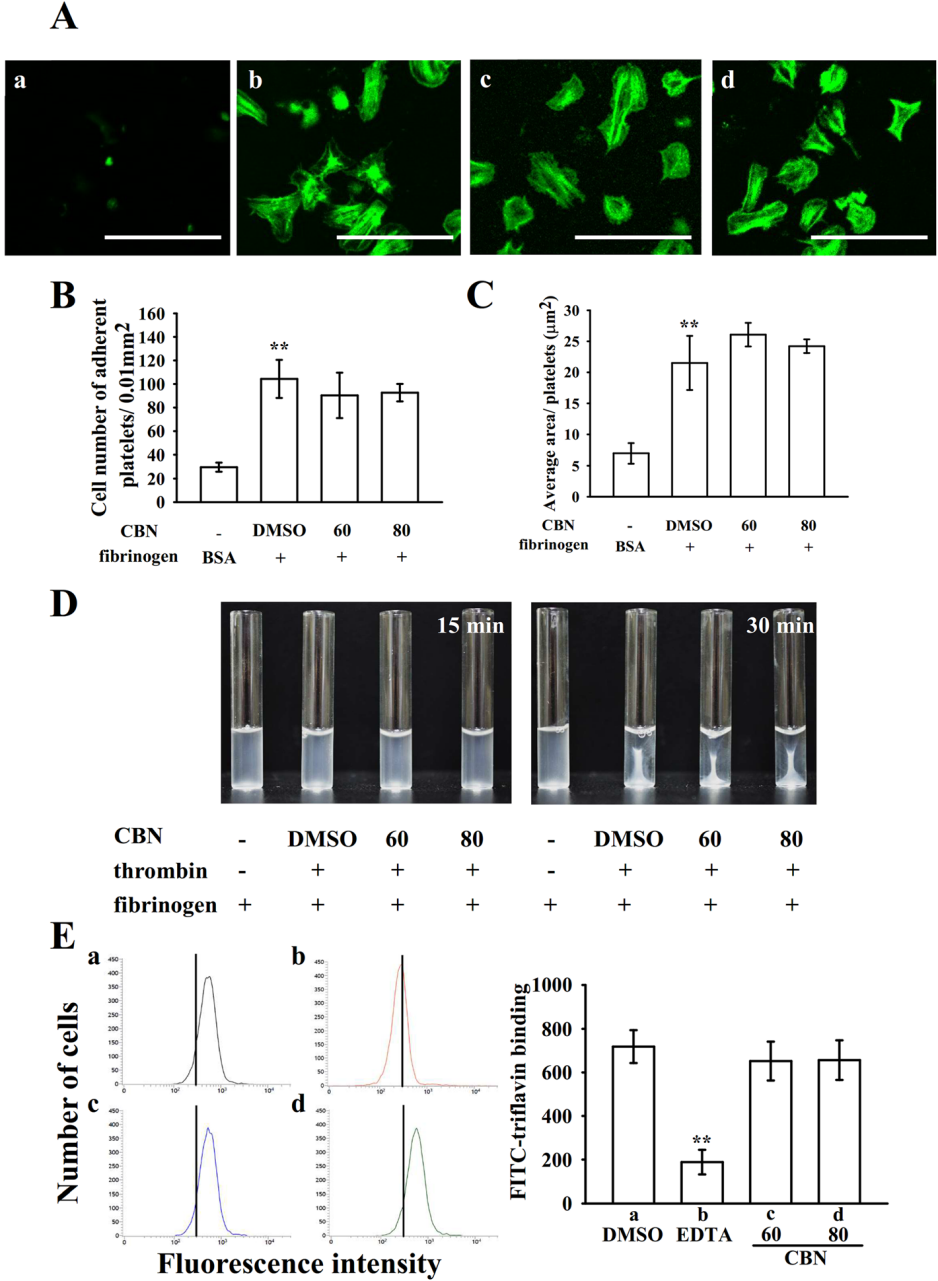
Effect of columbianadin (CBN) on platelet adhesion, spreading on immobilized fibrinogen and fibrin clot retraction as well as integrin α_IIb_β_3_ binding. (**a**) Washed platelets allowed to spread on the (*a*) BSA- or (*b*–*d*) fibrinogen-coated surfaces in the presence of the (*b*) solvent control (0.1% DMSO) or CBN (*c*, 60 μM; *d*, 80 μM) and subsequently labeled with FITC-conjugated phalloidin as described in the Materials and methods section. Plot of (**b**) the number of adherent platelets per 0.01 mm^2^ and (**c**) the average spreading surface area of individual platelets in six sight views. (**d**) Washed platelets suspended in 2 mg/mL fibrinogen with the solvent control (0.1% DMSO) or CBN (60 and 80 μM) before the thrombin (0.01 U/mL) stimulation. Images have been photographed at 15- and 30-min intervals. (**e**) For flow cytometry analysis, washed platelets were preincubated with solvent control (*a*, 0.1% DMSO, black line), EDTA (*b*, 2 mM, red line), and CBN (*c*, 60 μM, blue line; *d*, 80 μM, green line), followed by the addition of FITC-triflavin (2 μg/mL). Profiles in (**d**) are representative of four similar experiments. Data are presented as means ± SEM (*n* = 4). ^**^p < 0.01, compared with the immobilized BSA group (**b**, **c**) or 0.1% DMSO-treated group

Cyclic nucleotides such as cyclic AMP and cyclic GMP are critical secondary messengers that regulate multiple targets including different protein kinases, which have been reported to be involved in the phosphorylation of vasodilator-stimulated phosphoprotein (VASP). As illustrated in Fig. 5a, both ODQ (10 μM), a guanylate cyclase inhibitor and SQ22536 (100 μM), an adenylate cyclase inhibitor, significantly reversed NTG (10 μM)- and PGE_1_ (1 μM)-mediated inhibition of collagen-induced platelet aggregation, respectively. Neither ODQ nor SQ22536 significantly reversed CBN (80 μM)-mediated inhibition of platelet aggregation. Furthermore, both NTG (10 μM) and PGE_1_ (1 μM) obviously stimulated VASP phosphorylation, whereas CBN (60 and 80 μM) had no effects on cyclic nucleotide formation. Platelet aggregation is dependent on fibrinogen-integrin α_IIb_β_3_ interaction; nevertheless, integrin α_IIb_β_3_ inactivation can lead to disaggregation of aggregated platelets [17]. To further define whether CBN could disturb integrin α_IIb_β_3_ activation, the binding of the FITC-conjugated PAC-1 mAb that reacts with activation-induced conformational epitope of the integrin α_IIb_β_3_ was analyzed through flow cytometry. CBN treatment (60 and 80 μM) considerably affected integrin α_IIb_β_3_ activation stimulated by collagen, indicating that CBN may be interrupting the binding of activated integrin α_IIb_β_3_ (Fig. 5c). Furthermore, platelets adhered immobilized fibrinogen more significantly than immobilized bovine serum albumin (BSA) (Fig. 6a *a*–*b*), which was revealed by staining platelets with FITC-conjugated phalloidin. No significant differences were observed in platelet adhesion and spreading on immobilized fibrinogen for the CBN (60 and 80 μM)-treated platelets compared with the 0.1% DMSO-treated platelets (Fig. 6a *c*–*d*). As illustrated in Fig. 6b, control platelets were more predominantly fixed to immobilized fibrinogen compared with immobilized BSA (BSA, 29.7 ± 3.8 platelets/0.01 mm^2^ and fibrinogen, 104.3 ± 16.2 platelets/0.01 mm^2^; *n* = 4); however, both 60 and 80 μM CBN-treated platelets had similar adhesion to the fibrinogen-coated surface (60 μM, 90.3 ± 19.3 platelets/0.01 mm^2^, *n* = 4 and 80 μM, 92.7 ± 7.4 platelets/0.01 mm^2^, *n* = 4). Moreover, the surface coverage of a single platelet treated with CBN was not significantly different compared with 0.1% DMSO-treated platelets (0.1% DMSO, 21.5 ± 4.4 μm^2^; 60 μM, 26.1 ± 1.9 μm^2^ and 80 μM, 24.2 ± 1.1 μm^2^; *n* = 4) (Fig. 6c). Furthermore, clot retraction of fibrin polymers, the final step in thrombus formation, is essential for aggregate stabilization and a paradigm of integrin α_IIb_β_3_ outside-in signaling [1]. A clot retraction was performed by adding thrombin into a solution containing fibrinogen in the presence of CBN- or 0.1% DMSO-treated human platelets. As revealed in Fig. 6d, clot retraction was more apparent after 30-min incubation than that after 15-min incubation in 0.1% DMSO-treated platelets; however, no substantial decrease was observed in 60 and 80 μM CBN-treated platelets, indicating CBN had no significant ability to reduce fibrin clot retraction. Additionally, CBN did not attenuate phosphorylation of proteins, such as integrin β_3_, Src, and focal adhesion kinase, in platelets spreading on immobilized fibrinogen. For the purpose to further confirm that CBN did not directly affect integrin α_IIb_β_3_ binding, we performed a new study by using triflavin, an Arg-Gly-Asp-containing disintegrin purified from *Trimeresurus flavoviridis* venom, inhibits platelet aggregation through direct interference with fibrinogen binding to integrin α_IIb_β_3_ [8]. In particular, unlike the binding of fibrinogen or other disintegrins, which requires platelet activation, triflavin binds to resting and activated platelets with similar binding affinities (resting, Kd: 76.0 ± 9.6 nM vs. activated, Kd: 73.5 ± 7.4 nM) and binding numbers [18]. As shown in Fig. 6e, the relative intensity of FITC-triflavin (2 μg/mL) bound to resting platelets was 718.5 ± 75.5 (*a*, black line, *n* = 4), and it significantly decreased in the presence of 2 mM EDTA (negative control, 188.5 ± 56.5; *b*, red line, *n* = 4). CBN (60 and 80 μM) had no effects in reduction of FITC-triflavin binding (60 μM, 652.3 ± 89.2, *c*, blue line; 80 μM, 656.5 ± 91.1, *d*, green line; *n* = 4) in resting platelets. This result clearly rules out the possibility of CBN directly acts on integrin α_IIb_β_3_.

### Regulatory activities of CBN in integrin α_IIb_β_3_-mediated protein kinase activation and in vivo vascular thrombus formation

**Fig. 7:**
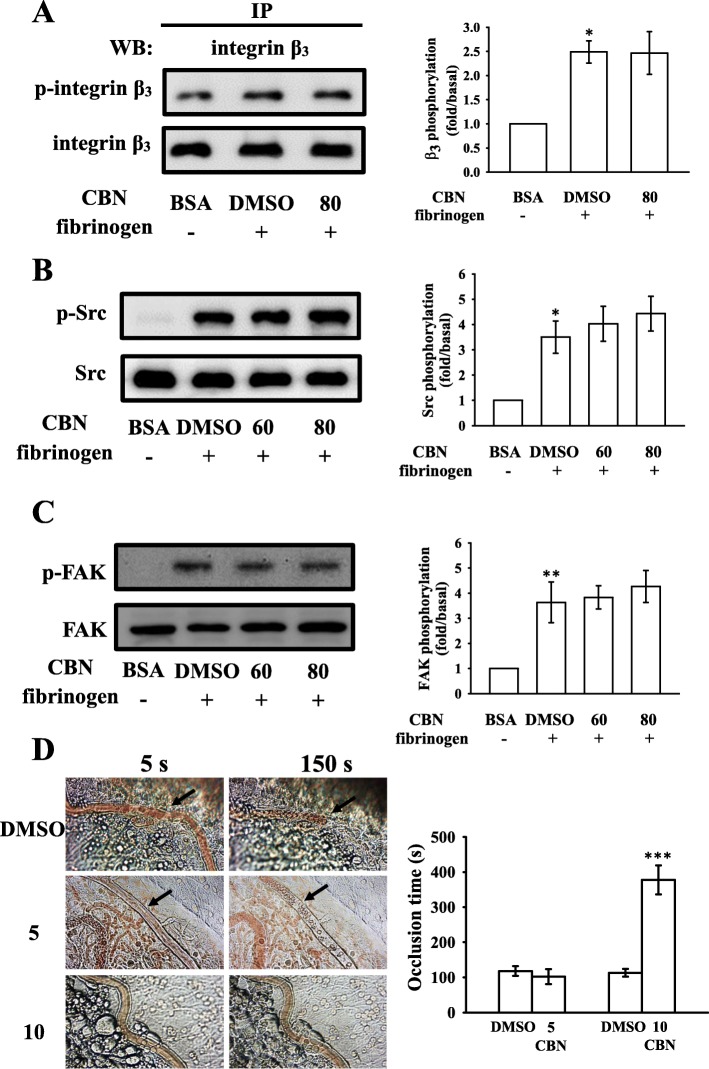
Effects of columbianadin (CBN) on integrin β_3_, Src, and FAK phosphorylation in platelets exposed to a fibrinogen-coated surface and on vascular thrombosis in the mesenteric venules of mice. (**a**) For immunoprecipitation study, washed platelets were preincubated with the solvent control (0.1% DMSO) or CBN (80 μM) and allowed to spread on immobilized fibrinogen (100 μg/mL). The platelets were lysed and Protein G Mag Sepharose Xtra beads were added with the anti-integrin β_3_ mAb (1 μg/mL) for immunoblotting. (**b**, **c**) Washed human platelets were preincubated with the solvent control (0.1% DMSO) or CBN (60 and 80 μM) and subsequently activated by immobilized fibrinogen (100 μg/mL) for determining the levels of (**b**) Src and (**c**) FAK phosphorylation. (**d**) For animal study, mice were administered an intravenous bolus of the solvent control (0.1% DMSO) or CBN (5 and 10 mg/kg), and the mesenteric venules were irradiated to induce microthrombus formation (occlusion time). Microscopic images (400× magnification) of 0.1% DMSO-treated controls and the 5 and 10 mg/kg CBN-treated groups were recorded at 5 and 150 s after irradiation, respectively. The photographs are representative of eight similar experiments, and the arrows indicate platelet plug formation. Data are presented as means ± SEM (**a**-**c**, *n* = 4; **d**, *n* = 8). ^*^p < 0.05 and ^**^p < 0.01, compared with the immobilized BSA group (**a**-**c**); ^***^p < 0.001, compared with the 0.1% DMSO-treated group (**d**)

To further elucidate the mechanisms by which CBN impairs integrin α_IIb_β_3_-mediated outside-in signaling, integrin β_3_ phosphorylation, a vital indicator of outside-in signaling, was studied. We examined integrin β_3_ phosphorylation in platelets exposed to immobilized fibrinogen through an immunoprecipitation assay and observed that integrin β_3_ phosphorylation was not significantly attenuated by CBN (80 μM) (Fig. 7a). CBN also had no significant effect on reversing immobilized fibrinogen-induced phosphorylation of Src and FAK (Fig. 7b–c). Overall these data suggested that CBN had no influence on integrin α_IIb_β_3_-mediated outside-in protein kinase phosphorylation.

The antithrombotic activity of CBN was observed in experimental mice. The occlusion time in the mesenteric microvessels of mice pretreated with 15 μg/kg fluorescein sodium was approximately 120 s. The resulting occlusion times were significantly extended after 5 and 10 mg/kg CBN treatments compared with those after 0.1% DMSO treatment (control vs. 5 mg/kg CBN, 118.0 ± 14.1 s vs. 113.1 ± 11.2 s, *n* = 8, *p* > 0.05; control vs. 10 mg/kg CBN, 102.3 ± 21.5 s vs. 377.7 ± 41.2 s, *n* = 8, *p* < 0.001; Fig. 7d). After irradiation, a thrombotic platelet plug was observed in the mesenteric microvessels at 5 and 150 s, in either 5 mg/kg CBN- or 0.1% DMSO-treated group (Fig. 7d; left panel, arrows). On administration of 10 mg/kg CBN, platelet plug formation was only observed at 5 s, but not at 150 s after irradiation (Fig. 7d). Furthermore, we also investigated and compared the therapeutic effects of CBN with aspirin in preventing acute pulmonary embolism death in mice as shown in Table 1. The results indicated that treatment with CBN at 5 and 10 mg/kg significantly lowered the ADP (0.7 mg/g)-induced mortality rate in mice from 100% (8 dead, *n* = 8) to 50% (4 dead, *n* = 8), and 0% (0 dead, *n* = 8), respectively. In addition, aspirin (20 mg/kg) also reduced the mortality to 25% (6 dead, *n* = 8) in this experiment (Table 1).

**Table 1:** Effect of columbianadin (CBN) and aspirin on mortality of acute pulmonary thrombosis caused by intravenous injection of ADP in experimental mice

	Total number	Number of deaths	Mortality (%)
ADP (0.7 mg/g)			
+ solvent control (0.1% DMSO)	8	8	100
+ CBN (5 mg/kg)	8	4	50
+ CBN (10 mg/kg)	8	0	0
+ aspirin (20 mg/kg)	8	2	25

## Discussion

This study reveals that in addition to the well-known properties of CBN, it also possesses antiplatelet activity in humans. It can be satisfactorily absorbed from gastrointestinal tract into bloodstream and distributed into organs [19]. Thus, the intake of CBN or natural of nontoxic prophylactic agents, such as food products and nutritional supplements, is ideal to prevent atherothrombotic events.

In the current study, CBN more potently inhibited collagen-induced platelet aggregation, but only slightly (not statistically significant) inhibited other platelet agonists; this implied that CBN was effective in inhibiting platelet aggregation through a prominent PLC-dependent mechanism. The platelet stimulation by agonists, for example collagen, noticeably modified phospholipase activation. The PLC activation resulted in IP_3_ and DAG formation, which activated PKC, inducing p47 protein phosphorylation [20]. PLC enzyme is composed of several subtypes in which PLCγ family can be further divided into two isozymes, namely PLCγ1 and PLCγ2. PLCγ2 participates in collagen-dependent signaling in platelets [21]. In our present study, CBN reduced the collagen-activated PLCγ2/PKC phosphorylation but without inhibition of PDBu-induced platelet aggregation; this suggested that CBN had no direct effects on PKC. Akt (downstream regulator of PI3K)-knockout mice have defective platelet activation [22]. Hence, Akt activation may be an attractive target for the development of antithrombotic therapeutics. Although effectors through which Akt contributes to platelet activation are not definitively known, several candidates have been discussed, including glycogen synthase kinase 3β, phosphodiesterase 3A, and the integrin β_3_ [22]. Additionally, it has been observed that both PI3K/Akt and MAPKs are mutually activated and PKC is the upstream regulator in platelets [23].

MAPKs constitute a family of serine/threonine kinases that convert extracellular stimuli into cellular responses. Conventional MAPKs can be divided into the ERK1/2, p38 MAPK (α, β, γ, and δ), JNK1/2, and big MAPK (ERK5) [24]. The ERK1/2, JNK1/2, and p38 MAPK have been found to participate in platelet activation [24]. All of these kinases are activated by specific MAPK kinases (MEKs). The intracellular roles of JNK1/2 and ERK1/2 in platelets remain unclear, but evidence shows that the suppression of integrin α_IIb_β_3_ activation may be involved [25]. Moreover, ERK activation is essential for collagen-induced platelet aggregation [26]. Cytosolic phospholipase A_2_ (cPLA_2_), which catalyzes AA release to produce thromboxane A_2_, which is an important substrate of p38 MAPK activation induced by various platelet agonists such as thrombin [27]. The present study revealed that CBN-mediated inhibition of collagen-stimulated platelet activation involved ERK1/2 and JNK1/2 activation, but not p38 MAPK activation, which may explain why CBN presents higher effectiveness for collagen stimulation than that for AA, U46619, and thrombin. Moreover, Fan et al. [24] reported that ERK1/2 and JNK1/2, but not p38 MAPK, are the major mitogen-activated protein 3 kinase (MEKK3) downstream signaling molecules in platelet activation. Therefore, we speculated that CBN may act on the MEKK3, resulting in inhibition of ERK1/2 and JNK1/2 phosphorylation. However, further studies are required for clarification.

Elevation of cyclic nucleotides, such as cyclic AMP and cyclic GMP, in platelets activates their respective protein kinase A and protein kinase G. This modulates platelet activation by phosphorylating intracellular protein substrates, such as VASP, which are involved in the inhibition of platelet aggregation and platelet adhesion [28]. Increased levels of cyclic nucleotides prevent most of the platelet responses and decrease the intracellular [Ca^2+^]i through Ca^2+^ uptake into the dense tubular system, which suppresses the activation of PLC/PKC signaling. In this study, neither SQ22536 nor ODQ significantly reversed the CBN-mediated inhibitory response, and CBN had no effects on VASP phosphorylation. Therefore, the CBN-mediated inhibition of platelet activation is independent of the intracellular cyclic nucleotides/VASP pathway.

The fibrinogen-integrin α_IIb_β_3_ binding belongs to a major component of activated platelets. Integrin α_IIb_β_3_ undergoes conformational changes upon activation, generating a unique and specific ligand-binding site for the fibrinogen, von Willebrand factor, and fibronectin [1]. Platelet adheres to immobilized fibrinogen and mediates clot retraction; these processes are involved in integrin α_IIb_β_3_ outside-in signaling [1]. PAC-1 reacts with the activation-induced conformational epitope of integrin α_IIb_β_3_ [29], and the PAC-1 binding was observed to be markedly reduced by CBN treatment. In addition, platelet-mediated fibrin clot retraction is also mediated by integrin α_IIb_β_3_. Integrin α_IIb_β_3_-mediated signaling begins immediately after a fibrinogen molecule binds to the integrin; this outside-in signaling results in tyrosine phosphorylation of numerous proteins, such as the Src family kinases (SFK; e.g., Src, Lyn, and Fyn), FAK, and the cytoplasmic tail of integrin β_3_ at Tyr759, a process dependent on outside-in signaling and cytoskeleton reorganization [1]. The critical role of integrin β_3_ at Tyr759 in platelets was demonstrated in vivo, and its mutation led to bleeding disorder and strongly affected clot retraction responses in vitro [30]. FAK, a cytoplasmic tyrosine kinase located at focal adhesion points, plays a vital role in cytoskeleton regulation and integrin α_IIb_β_3_ activity [31]. Platelet adhesion to immobilized fibrinogen requires FAK activation through integrin α_IIb_β_3_, and in turn activation of FAK requires autophosphorylation [31]. In this study, CBN had no effects on platelet adhesion and spreading and fibrin clot retraction, as well as phosphorylation of integrin β_3_, Src, and FAK on immobilized fibrinogen, indicating that CBN influences integrin α_IIb_β_3_ inside-out but not outside-in signaling.

After vascular endothelial cell injury, exposure to subendothelial collagen is the major trigger that initiates platelet adhesion and aggregation at the injury site, followed by vascular thrombosis. Animal models of vascular thrombosis are necessary in order to understand the effectiveness of test compounds for this disease. An ideal mouse model should be technically simple, quick in operation, and easily reproducible. In a vascular thrombotic mice model [11], mesenteric venules were continuously irradiated by fluorescein sodium throughout the experimental period, leading to strong damage to the endothelium, treatment with 10 mg/kg CBN significantly extended the occlusion times; in studies on acute pulmonary thromboembolism, platelet aggregation is intimately involved in experimental thrombosis, and CBN effectively prevented ADP-induced thromboembolic death. We also found that CBN is more effectiveness than aspirin at lowering mortality in acute pulmonary thromboembolism. These data are consistent with the fact that platelet aggregation is a more crucial factor causing vascular thrombosis. Therefore, CBN may represent a potential natural compound for treating thromboembolic disorders.

## Conclusion

This study revealed a novel role of CBN in the inhibition of platelet activation in humans, suggesting that it can be used for potential therapeutic or prophylactic applications. The outcome of this study may provide a new insight into the role of CBN in human platelet activation because it significantly inhibited platelet activation by hindering the PLCγ2-PKC cascade and subsequently, suppressed the activation of Akt and ERKs/JNKs. These changes decrease the release, such as [Ca^2+^]i, followed by integrin α_IIb_β_3_ inside-out signaling and inhibition of platelet aggregation.

## Data Availability

All data generated or analyzed during this study are included in this published article.

## Abbreviations

AA: Arachidonic acid
ADP: Adenosine diphosphate
ATP: Adenosine triphosphate
BSA: Bovine serum albumin
CBN: Columbianadin
CVDs: Cardiovascular diseases
DAG: Diacylglycerol
DMSO: Dimethyl sulfoxide
ERK: Extracellular signal-regulated kinase
FAK: Focal adhesion kinase
IP_3_: Inositol trisphosphate
JNK: c-Jun N-terminal kinase
LDH: Lactate dehydrogenase
LPS: Lipopolysaccharide
MAPK: Mitogen-activated protein kinase
NO: Nitric oxide
NTG: Nitroglycerin
PDBu: Phorbol-12,13-dibutyrate
PGE_1_: Prostaglandin E_1_
PKC: Protein kinase C
PLCγ2: Phospholipase Cγ2
VASP: Vasodilator-stimulated phosphoprotein
